# Grazing Is Associated With Dietary Diversity and Gastrointestinal Microbiota in Subterranean Rodents

**DOI:** 10.1002/ece3.72377

**Published:** 2025-11-02

**Authors:** Shien Ren, Jing Yang, Xiaoluo Aba, Yang Hu, Yifan Zhao, Shoushu Pang, Chongxuan Han, Liangzhi Zhang, Xiaoning Nan

**Affiliations:** ^1^ Key Laboratory of National Forestry and Grassland Administration on Management of Western Forest Bio‐Disaster, College of Forestry Northwest A&F University Yangling Shaanxi China; ^2^ Institute of Bailongjiang Forestry Science of Gansu Province Lanzhou China; ^3^ Key Laboratory of Adaptation and Evolution of Plateau Biota, Northwest Institute of Plateau Biology Chinese Academy of Sciences Xining China

**Keywords:** community assembly, dietary diversity, gastrointestinal microbiota, grazing, plateau zokor

## Abstract

Diet significantly influences gut microbiota composition. Grazing activities alter local vegetation communities, which in turn are related to changes in the availability of food resources for wildlife. However, the relationships between grazing and the dietary diversity and gut microbiota in subterranean rodents remain poorly understood. Using the plateau zokor (*Eospalax baileyi*) as a model species, chloroplast *trnL* and 16S rRNA gene sequencing were employed to characterize dietary composition and gastrointestinal microbial communities in zokors from grazing and non‐grazing (control) areas. The analysis revealed significant differences in dietary diversity and composition of zokors between grazing and non‐grazing areas. Meanwhile, the gastrointestinal microbial community diversity differed significantly between groups. Beneficial microbes (e.g., Lachnospiraceae and Christensenellaceae) showed higher abundance in the control group, while pathogenic Proteobacteria were enriched in the grazing groups. Notably, the complexity of stomach microbial co‐occurrence networks increased in the grazing group, and functional differentiation of the gastrointestinal microbiota was observed between the control and grazing groups. Community assembly analysis displayed that stochastic processes dominated the assembly of stomach and gut microbiota, though these processes diminished in the grazing group, where divergent ecological processes emerged. Furthermore, we identified robust associations between zokor diets and stomach/gut microbiota, with specific microbial taxa significantly correlated with particular plants. Collectively, these findings highlight that grazing is associated with dietary changes and gut microbiota shifts in subterranean rodents, thereby advancing our understanding of the ecological impacts of grazing on terrestrial ecosystems.

## 1 Introduction

The gastrointestinal microbiota serves essential functions in host nutrient metabolism, immune regulation, and environmental adaptation, with its composition and function influenced by host diet, ecological environment, and behavioral strategies (Flint et al. 2012; Gacesa et al. 2022; Li, Li, Li, et al. 2019; Li, Li, Kohl, et al. 2019; Martinez‐Mota et al. 2020; Maslowski et al. 2009; Moeller et al. 2016; Ren et al. 2024). Diet represents a fundamental factor in shaping gut microbiota composition and functional dynamics, as dietary modifications can trigger rapid and reproducible alterations in gut microbial community structures (David et al. 2014). Current research indicates that dietary diversity fundamentally drives microbial community dynamics by determining the range of available substrates for microbes, thus influencing functional redundancy and niche differentiation within microbial ecosystems (Carmody et al. 2015; David et al. 2014; Li et al. 2023). Enhanced dietary diversity correlates positively with increased gut microbial diversity, while promoting beneficial microbial taxa and reducing potential pathogen abundance, thereby strengthening the host's environmental adaptation capacity (Heiman and Greenway 2016; Sonnenburg et al. 2016; Xiao et al. 2022). Nevertheless, the relationship between dietary diversity and gut microbiota in subterranean rodents remains inadequately understood.

Traditional microhistological analysis, though commonly employed in animal dietary studies, presents limitations including low accuracy and dependence on expert assessment (Shan and Wu 2005). DNA metabarcoding technology has recently enabled noninvasive and high‐resolution dietary analysis, particularly effective in identifying complex plant fragments while accommodating large‐scale sample processing (Ando et al. 2020; Pompanon et al. 2012; Ruppert et al. 2019). The *trnL* approach stands as a crucial method for characterizing herbivore diets through metabarcoding, capable of identifying approximately 50% of plant taxa to species level from fecal samples, accurately reflecting herbivore dietary composition (Valentini et al. 2009). This methodology has found widespread application in herbivore dietary research (Cao et al. 2024; He et al. 2025; Kartzinel and Pringle 2020; Nielsen and Matocq 2021; Pansu et al. 2022; Zhou et al. 2024). Zhang, Zou, et al. (2022) initially applied the *trnL* method to examine Gansu zokor and Smith's zokor diets, demonstrating DNA metabarcoding's applicability for studying subterranean rodents that exclusively inhabit underground environments and primarily consume plant roots and tubers. Thus, DNA metabarcoding technology facilitates more precise and efficient assessment of herbivore dietary diversity, enabling deeper investigation of dietary diversity–gut microbiota relationships.

Grazing constitutes the primary production activity on the Qinghai–Tibet Plateau and functions as a crucial ecological factor influencing grassland ecosystem structure and function. Moderate grazing typically supports ecosystem stability and service functions. Research indicates that appropriate grazing can enhance grassland species diversity while inhibiting exotic species invasion (Li et al. 2021; Wang et al. 2020). However, overgrazing reduces vegetation cover, increases soil exposure, and accelerates erosion and grassland degradation (Hanke et al. 2014; Porensky et al. 2020; Souther et al. 2019). Grazing affects community structure both directly through plant consumption and indirectly by modifying ecological factors such as soil properties, snow accumulation, and spring soil moisture content, thereby influencing vegetation spatial distribution and community succession (Yan et al. 2019). These vegetation community alterations may affect wild animal dietary selection, subsequently modifying their gut microbiota. Therefore, elucidating the relationship between grazing and dietary diversity and gut microbiota of sympatric wild animals has become increasingly critical.

The plateau zokor (*Eospalax baileyi*), an endemic subterranean rodent of the Qinghai–Tibet Plateau, maintains an extensive regional distribution. This species creates foraging tunnels through burrowing activities to access plant roots and rhizomes. Grazing may modify surface vegetation community structure and underground root system distribution, thereby affecting zokor food resource availability. This investigation combines DNA metabarcoding technology and high‐throughput sequencing methods to examine: (1) grazing's impact on zokor dietary diversity; (2) grazing's influence on zokor gut microbiota; and (3) the relationship between dietary diversity and gut microbiota in zokors. These findings will advance our understanding of grazing effects on alpine grassland ecosystems.

## 2 Materials and Methods

### 2.1 Study Area

The study area is located in the alpine meadows of Zhuoni County, Gansu Province, on the northeastern margin of the Qinghai–Tibet Plateau (Figure S1). The region has a plateau continental climate, with an average annual temperature of 5.6°C, a maximum temperature of 33.5°C, and a minimum temperature of −19.2°C. The mean annual sunshine duration is 2450.4 h, the average annual precipitation is 487.1 mm, and the frost‐free period lasts approximately 114 days. The dominant soil types are alpine meadow soils, corresponding to Cambisols in the World Reference Base for Soil Resources classification system (Sorokin et al. 2021). At all sampling sites, the plateau zokor is the only subterranean rodent inhabiting the alpine meadow ecosystem. The grazing area is a warm‐season grazing zone, with yaks and Tibetan sheep grazing during the sampling period. The control area is a cold‐season grazing zone, which was fenced off for conservation from mid‐April to early October, with no livestock grazing during the sampling period.

### 2.2 Sample Collection

Plateau zokors were captured during June in the study area. Nine individuals were captured from grazing areas. Specifically, three 1‐ha plots were established in the grazing areas, with each plot spaced 2 km apart. Three adult zokors were captured per plot. Using the same method, nine zokors were captured in non‐grazing areas as a control group (Table S1). Immediately after capture, the zokors were humanely euthanized in accordance with ethical guidelines. Stomach and gut (cecal) contents were extracted, preserved in 2 mL cryovials, and flash‐frozen in liquid nitrogen. Stomach and gut samples from the control area were designated as SC and GC groups, while those from the grazing area were labeled SG and GG groups, respectively. All experimental procedures were approved by the Ethics Committee of Northwest A&F University and adhered to institutional animal welfare regulations.

### 2.3 DNA Extraction, PCR Amplification, and Illumina Sequencing

Total genomic DNA was extracted from stomach and gut content samples using the QIAamp DNA Stool Mini Kit (Qiagen, Germany) following the manufacturer's protocol. DNA quality and concentration were assessed by 1.0% agarose gel electrophoresis and a NanoDrop 2000 spectrophotometer (Thermo Fisher Scientific, USA), after which the samples were stored at −80°C until further analysis. For dietary analysis, the chloroplast *trnL* gene was amplified from stomach contents using the primer pair *trnL*‐c‐F (5′‐CGAAATCGGTAGACGCTACG‐3′) and *trnL*‐h‐R (5′‐CCATTGAGTCTCTGCACCTATC‐3′). To characterize the gastrointestinal microbiota, the V3–V4 hypervariable region of the 16S rRNA gene was amplified from both stomach and gut contents using the primers 338F (5′‐ACTCCTACGGGAGGCAGCAG‐3′) and 806R (5′‐GGACTACHVGGGTWTCTAAT‐3′).

The PCR reaction mixture (20 μL total volume) consisted of 4 μL of 5× Fast Pfu buffer, 2 μL of 2.5 mM dNTPs, 0.8 μL of each primer (5 μM), 0.4 μL Fast Pfu polymerase, 10 ng of template DNA, and nuclease‐free water. Amplification was performed under the following conditions: initial denaturation at 95°C for 3 min; 30 cycles of denaturation (95°C, 30 s), annealing (55°C, 30 s), and extension (72°C, 45 s), followed by a final extension at 72°C for 10 min and cooling to 4°C. PCR products were separated on a 2% agarose gel, purified using a PCR Clean‐Up Kit (Yuhua, China), and quantified with a Qubit 4.0 fluorometer (Thermo Fisher Scientific, USA). Finally, purified amplicons were sequenced on an Illumina MiSeq platform (Illumina, USA) by a commercial biotechnology company.

### 2.4 Bioinformatics and Statistical Analysis

Raw FASTQ files were demultiplexed using an in‐house Perl script, followed by quality filtering with fastp (v0.19.6) (Chen et al. 2018) and merging using FLASH (v1.2.7) (Magoc and Salzberg 2011). The processed sequences were clustered into operational taxonomic units (OTUs) at a 97% similarity threshold using UPARSE (v7.1) (Edgar 2013), with the most abundant sequence selected as the representative sequence for each OTU. For dietary analysis, the QIIME (v1.91) feature‐classifier plugin aligned OTU sequences against the National Center for Biotechnology Information database for taxonomic classification and taxonomy table generation. For microbial analysis, the OTU table was manually filtered to remove chloroplast and mitochondrial sequences. Representative OTU sequences were taxonomically classified using the RDP Classifier (v2.2) (Wang et al. 2007) against the SILVA (v138) reference database (https://www.arb‐silva.de) with a confidence threshold of 0.7. Finally, metagenomic functions were predicted using PICRUSt2 (Douglas et al. 2020) based on OTU representative sequences aligned against the Kyoto Encyclopedia of Genes and Genomes (KEGG) database (https://www.kegg.jp).

Alpha diversity indices, including the Shannon and Invsimpson indices, were calculated from OTU data using Mothur v1.30.1 (Schloss et al. 2009). Venn diagrams were generated with Venny 2.1.0 (https://bioinfogp.cnb.csic.es/tools/venny/index.html). Principal coordinate analysis (PCoA) was conducted using the “vegan” v2.5‐6 package (Dixon 2003) based on Bray–Curtis dissimilarity matrices of dietary and microbial community composition. Permutational multivariate analysis of variance (PERMANOVA) was applied to evaluate the proportion of variation attributable to treatment effects, along with statistical significance. Linear discriminant analysis effect size (LEfSe) (http://huttenhower.sph.harvard.edu/LEfSe) was employed to identify significantly enriched KEGG categories across groups (LDA score > 2.5, *p* < 0.05). Spearman's correlation analysis of the top 50 bacterial OTUs in each group was performed using the “psych” package (Yuen et al. 2018), with co‐occurrence events considered statistically significant at |*R*| > 0.5 and *p* < 0.05. The resulting network was visualized in Gephi v0.9.2 (Jacomy et al. 2014). The neutral community model was fitted using the “Hmisc” package. The normalized stochasticity ratio (NST) was computed with the “NST” package to quantify the relative contributions of stochastic and deterministic processes in microbial community assembly, using a 50% threshold to determine dominance (Ning et al. 2019). Additionally, the phylogenetic bin‐based null model analysis (iCAMP) was applied to evaluate the relative importance of different ecological processes in gut microbial assembly (Ning et al. 2020). Differences in dietary diversity, dietary composition, microbial diversity, microbial composition, network parameters, NST values, and ecological processes between groups were evaluated using the Wilcoxon rank‐sum test. Linear regression and Procrustes analysis were used to examine relationships between dietary clusters and microbial communities. Finally, associations between dominant dietary components and microbial composition were assessed using the “pheatmap” package. Statistical significance was set at a *p* < 0.05 after the Benjamini–Hochberg false discovery rate correction (Benjamini and Hochberg 1995).

## 3 Results

### 3.1 Diet Diversity and Composition of Zokors

**FIGURE 1:**
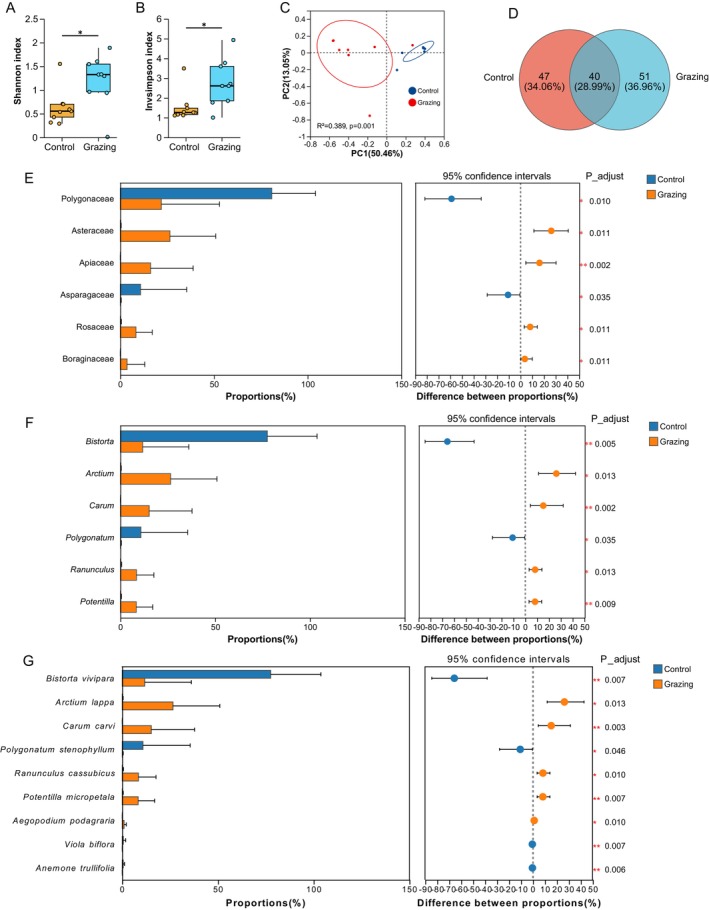
Differences in diet diversity and composition of zokors. (A) Shannon index, (B) Invsimpson index, (C) PCoA analysis, (D) Venn diagram, (E) Differential families, (F) Differential genera, and (G) Differential species. *p < 0.05, **p < 0.01, and ***p < 0.001.

A total of 990,052 high‐quality clean paired reads were obtained from 18 stomach samples, which were taxonomically classified into 2 phyla, 5 classes, 21 orders, 28 families, 57 genera, and 68 species. Both the Shannon and Invsimpson indices were significantly higher in the grazing group compared to the control group (*p* < 0.05) (Figure 1A,B). PCoA results based on Bray–Curtis dissimilarity revealed distinct clustering of dietary communities between the grazing and control groups (PERMANOVA, *R* ^2^ = 0.389, *p* = 0.001) (Figure 1C). The Venn diagram illustrated 51 (36.96%) unique OTUs in the grazing group, 47 (34.06%) unique OTUs in the control group, and 40 (28.99%) shared OTUs between the two groups (Figure 1D).

The plant species consumed by plateau zokors primarily belong to the families Polygonaceae, Asteraceae, and Apiaceae. At the genus level, the dominant genera include *Bistorta*, *Arctium*, *Carum*, *Cyamopsis*, *Polygonatum*, *Ranunculus*, *Potentilla*, *Rumex*, and *Carex*. At the species level, the most frequently consumed species are *Bistorta vivipara* , *Arctium lappa* , *Carum carvi* , *Cyamopsis tetragonoloba* , *Polygonatum stenophyllum*, *Ranunculus cassubicus*, *Potentilla micropetala*, *Rumex alpinus* , *Carex maritima* , and *Koenigia nepalensis* (Figure S2). Intergroup difference analysis revealed significant variations in relative abundances between the grazing and control groups. At the family level, Asteraceae, Apiaceae, Rosaceae, and Boraginaceae were significantly more abundant in the grazing group (*p* < 0.05), whereas Polygonaceae and Asparagaceae were significantly less abundant (*p* < 0.05) (Figure 1E; Table S2). Similarly, at the genus level, *Arctium*, *Carum*, *Ranunculus*, and *Potentilla* exhibited higher relative abundances in the grazing group (*p* < 0.05), while *Bistorta* and *Polygonatum* were significantly reduced (*p* < 0.05) (Figure 1F; Table S2). At the species level, *Arctium lappa* , *Carum carvi* , *Ranunculus cassubicus*, *Potentilla micropetala*, and *Aegopodium podagraria* were more prevalent in the grazing group (*p* < 0.05), whereas *Bistorta vivipara* , *Polygonatum stenophyllum*, *Viola biflora* , and *Anemone trullifolia* were less abundant (*p* < 0.05) (Figure 1G; Table S2).

### 3.2 Diversity and Composition of Gastrointestinal Microbiota in Zokors

**FIGURE 2:**
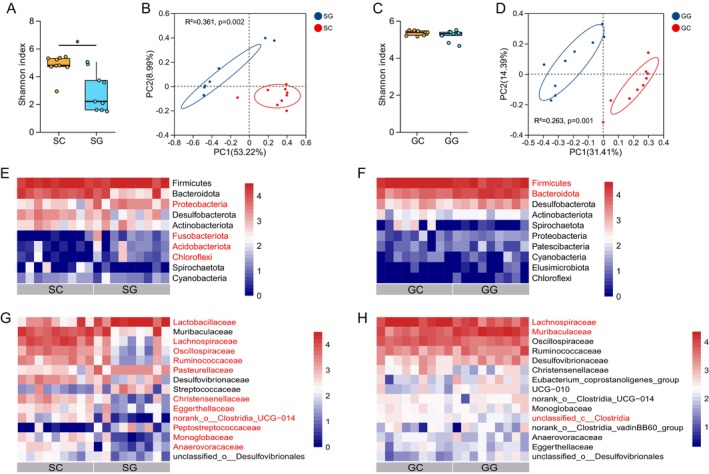
Differences in microbial diversity and composition in the stomach and gut of zokors. (A) Shannon index and (B) PCoA analysis of stomach microbiota. (C) Shannon index and (D) PCoA analysis of gut microbiota. Differential (E) phyla and (G) families of stomach microbiota. Differential (F) phyla and (H) families of gut microbiota. Red font represents microbial taxa with significant differences between groups. *p < 0.05.

The Shannon index of stomach microbiota was significantly lower in the SG group compared to the SC group (*p* < 0.05), whereas no significant difference was observed between the GG and GC groups in gut microbiota diversity (Figure 2A,C). PCoA analysis based on Bray–Curtis dissimilarity revealed distinct clustering of stomach microbiota between the SG and SC groups (PERMANOVA, *R* ^2^ = 0.361, *p* = 0.002), as well as significant separation of gut microbiota between the GG and GC groups (PERMANOVA, *R* ^2^ = 0.263, *p* = 0.001) (Figure 2B,D). Venn diagram analysis revealed 1310 (24.90%) unique OTUs in the SG group and 2504 (47.60%) unique OTUs in the SC group for stomach microbiota, with 1447 (27.50%) OTUs shared between the two groups. In the gut microbiota, the GG group contained 1985 (30.57%) unique OTUs, the GC group had 2317 (35.68%) unique OTUs, and the two groups shared 2192 (33.75%) OTUs (Figure S3).

The predominant bacterial phyla identified in the stomach microbiota of zokors were Firmicutes, Bacteroidota, and Proteobacteria, with the dominant families comprising Lactobacillaceae, Muribaculaceae, Lachnospiraceae, Oscillospiraceae, and Ruminococcaceae (Figure S4). Comparative analysis revealed significant differences at the phylum level: Proteobacteria, Fusobacteriota, Acidobacteriota, and Chloroflexi exhibited significantly higher relative abundances in the SG group compared to the SC group (*p* < 0.05) (Figure 2E; Table S3). At the family level, Lactobacillaceae, Pasteurellaceae, and Peptostreptococcaceae were more abundant in the SG group, whereas Lachnospiraceae, Oscillospiraceae, Ruminococcaceae, Christensenellaceae, Eggerthellaceae, norank_o__Clostridia_UCG‐014, Monoglobaceae, and Anaerovoracaceae showed significantly higher abundances in the SC group (*p* < 0.05) (Figure 2G; Table S3). In the gut microbiota, Firmicutes, Bacteroidota, and Desulfobacterota emerged as the dominant phyla, with Lachnospiraceae, Muribaculaceae, Oscillospiraceae, Ruminococcaceae, and Desulfovibrionaceae representing the predominant families (Figure S5). At the phylum level, the GG group displayed significantly elevated abundances of Bacteroidota relative to the GC group, while Firmicutes abundance was notably reduced (*p* < 0.05) (Figure 2F; Table S4). At the family level, Muribaculaceae was more abundant in the GG group, whereas Lachnospiraceae and unclassified_c__Clostridia were significantly less abundant compared to the GC group (*p* < 0.05) (Figure 2H; Table S4).

### 3.3 Co‐Occurrence Network of Gastrointestinal Microbiota in Zokors

**FIGURE 3:**
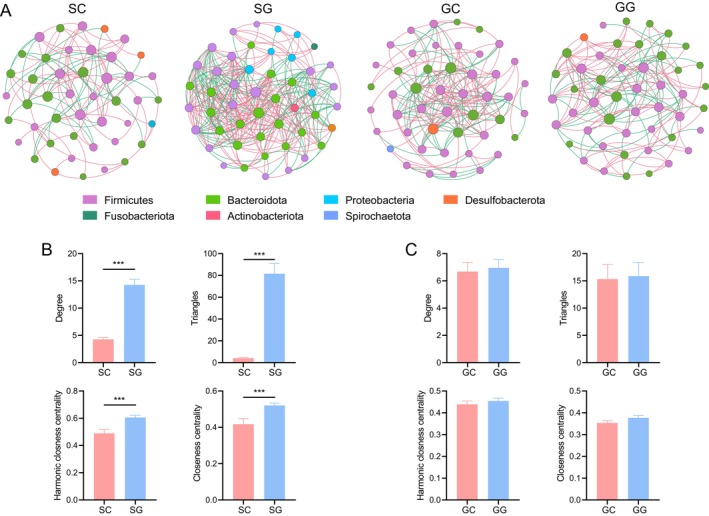
Microbial co‐occurrence network in the stomach and gut of zokors. (A) Co‐occurrence network. Node‐level topological features of the co‐occurrence network in the (B) stomach and (C) gut. ***p < 0.001.

Co‐occurrence network analysis highlighted complex interactions within the gastrointestinal microbiota of zokors (Figure 3A). In the stomach microbiota, the SG group exhibited 48 nodes and 342 links, whereas the SC group contained 45 nodes and 96 links (Table S5). Node‐level topological parameters were calculated for both groups, revealing significantly higher values for degree, triangles, harmonic closeness centrality, and closeness centrality in the SG group compared to the SC group (*p* < 0.001) (Figure 3B). In the gut microbiota, the GG group comprised 48 nodes and 167 links, while the GC group consisted of 47 nodes and 157 links (Table S5). However, no significant differences in node‐level topological parameters were observed between the GG and GC groups (Figure 3C).

### 3.4 Functional Profile of Gastrointestinal Microbiota in Zokors

**FIGURE 4:**
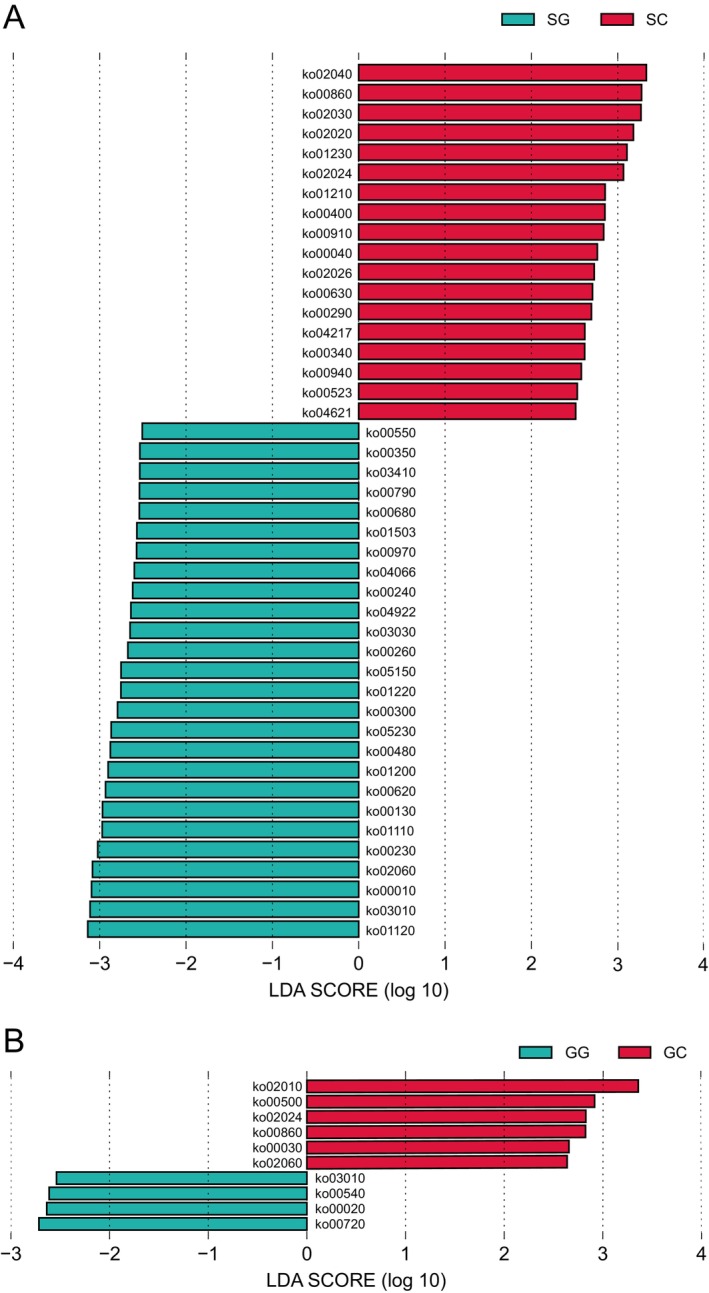
Differences in microbial metabolic categories in zokors. Alterations in microbial metabolic categories at KEGG level 3 in the (A) stomach and (B) gut identified by LEfSE analysis with LDA > 2.5, *p* < 0.05.

We employed LEfSe analysis to identify functional differences in the zokor gastrointestinal microbiota between groups. In the stomach microbiota, the SG group exhibited enrichment of 26 KEGG level‐3 pathways, primarily categorized under the level‐2 pathways Global and overview maps (ko01220, ko01200, ko01110, and ko01120), Amino acid metabolism (ko00350, ko00260, and ko00300), and Carbohydrate metabolism (ko00620 and ko00010). In contrast, the SC group showed enrichment of 18 level‐3 pathways, primarily assigned to Amino acid metabolism (ko00400, ko00290, and ko00340), Carbohydrate metabolism (ko00040 and ko00630), and Global and overview maps (ko01230 and ko01210) (Figure 4A; Table S6). In the gut microbiota, four level‐3 pathways (ko03010, ko00540, ko00020, and ko00720) were enriched in the GG group, while six pathways (ko02010, ko00500, ko02024, ko00860, ko00030, and ko02060) were enriched in the GC group (Figure 4B; Table S7).

### 3.5 Assembly Process of Gastrointestinal Microbiota in Zokors

**FIGURE 5:**
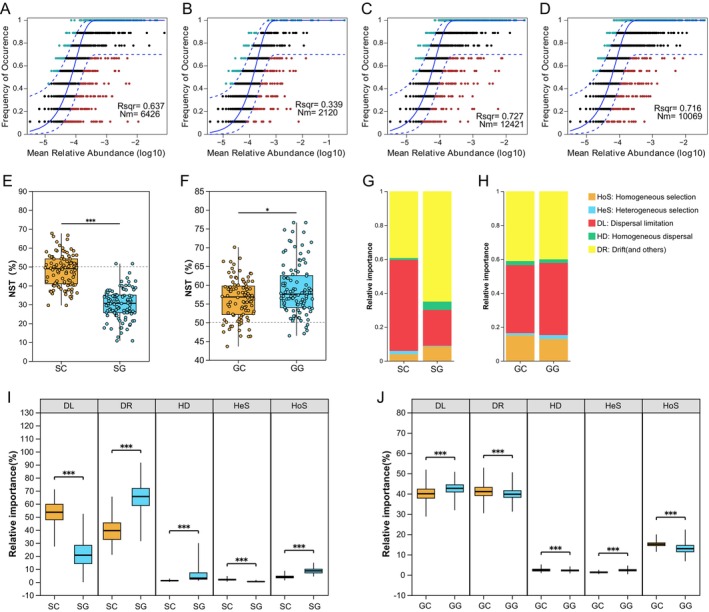
Assembly process of microbial communities in the stomach and gut of zokors. Fit of the neutral community model (NCM) in the (A) SC, (B) SG, (C) GC, and (D) GG groups. Solid lines represent the fitting of the neutral model, while the upper and lower dashed lines indicate the 95% confidence bounds predicted by the model. OTUs that occur more or less frequently than predicted by the NCM are shown in different colors. Nm represents the metacommunity size times immigration, *R* ^2^ indicates the fit to this model. Normalized stochasticity ratio of microbial communities in the (E) stomach and (F) gut. Ecological processes involved in the gut microbial community assembly and their difference between groups in the (G, I) stomach and (H, J) gut. *p < 0.05 and ***p < 0.001.

The NCM was employed to elucidate the potential importance of stochastic processes in the assembly of gastrointestinal microbial communities in zokors. The results indicated that the NCM effectively explained most relationships between OTU occurrence frequencies and their relative abundance variations. For stomach microbiota, the community variance explained was 63.7% in the SC group and 33.9% in the SG group, with higher microbial taxon Nm values observed in the SC group (Nm = 6426) compared to the SG group (Nm = 2120) (Figure 5A,B). In the gut microbiota, the explained community variance reached 72.7% in the GC group and 71.6% in the GG group, while microbial taxa exhibited higher Nm values in the GC group (Nm = 12,421) than in the GG group (Nm = 10,069) (Figure 5C,D). These findings suggest that the dispersal capacity mediated by stochastic processes in the zokor stomach and gut microbiota was attenuated under grazing conditions.

We additionally performed NST analysis to quantify the roles of deterministic and stochastic processes in shaping gastrointestinal microbial communities. For stomach microbiota, the SC group showed a significantly higher mean NST value (47.88%) compared to the SG group (30.29%) (*p* < 0.001; Figure 5E), indicating that microbial assembly in the SG group was predominantly governed by deterministic processes. In the gut microbiota, the GC group exhibited a significantly lower mean NST value (56.27%) than the GG group (58.81%) (*p* < 0.05; Figure 5F), though both groups remained primarily influenced by stochastic processes.

Further quantitative assessment using phylogenetic bin‐based null model analysis revealed the dominant role of stochastic processes (including dispersal limitation, homogenizing dispersal, and drift) in microbial community assembly. These processes accounted for 94.12% in the SC group and 91.10% in the SG group of stomach microbiota assembly, and 83.61% in the GC group and 84.75% in the GG group of gut microbiota assembly (Figure 5G,H). Statistical analyses revealed significant differences in all ecological processes between treatment groups for both stomach and gut microbiota (*p* < 0.001) (Figure 5I,J), suggesting the role of grazing in driving the formation of the gastrointestinal microbiota in zokors.

### 3.6 Association Between Diet and Gastrointestinal Microbiota in Zokors

**FIGURE 6:**
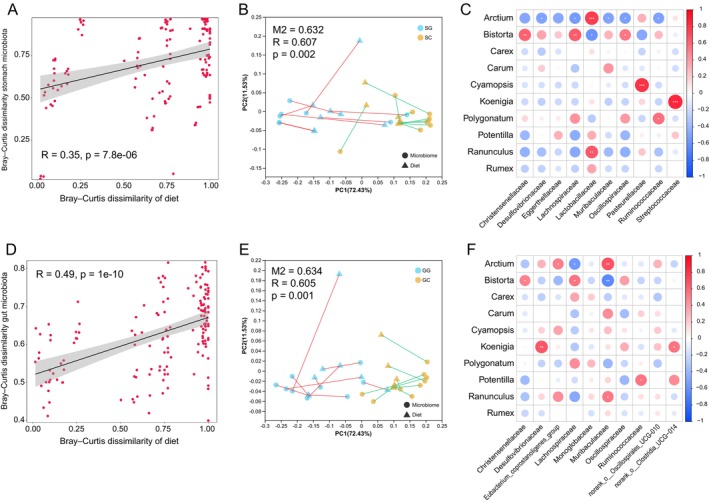
The relationship between zokor diet and stomach/gut microbiota. Linear regression between dietary beta diversity and (A) stomach and (D) gut microbial beta diversity. Procrustes analysis between diet and (B) stomach and (E) gut microbiota. Spearman's correlation heatmap between specific dietary plant genera and (C) stomach and (F) gut microbes. *p < 0.05, **p < 0.01, and ***p < 0.001.

Linear regression analysis identified significant positive correlations between dietary beta diversity and both stomach (*R* = 0.35, *p* < 0.001) and gut (*R* = 0.49, *p* < 0.001) microbial beta diversity in zokors (Figure 6A,D). Procrustes analysis was applied to assess the concordance between microbial community structure ordinations and dietary composition ordinations, revealing significant congruence between the two (Stomach, M^2^ = 0.632, *R* = 0.607, *p* = 0.002; Gut, M^2^ = 0.634, *R* = 0.605, *p* = 0.001) (Figure 6B,E). This further confirmed a strong association between dietary composition and the structure of stomach and gut microbial communities. Correlation heatmap analysis highlighted specific relationships between microbes and plants. For instance, in the stomach, the plant *Bistorta* showed significant positive correlations with Anaerovoracaceae, Christensenellaceae, Lachnospiraceae, and Oscillospiraceae; *Cyamopsis* was significantly positively correlated with Pasteurellaceae; and *Koenigia* exhibited a significant positive correlation with Streptococcaceae. Conversely, *Arctium* displayed significant negative correlations with nine bacterial families (Figure 6C). In the gut, *Arctium* was significantly positively correlated with Muribaculaceae and Eubacterium_coprostanoligenes_group, but negatively correlated with Lachnospiraceae and unclassified_c__Clostridia; *Koenigia* showed significant positive correlations with Desulfovibrionaceae, norank_o__Clostridia_UCG–014, and unclassified_o__Desulfovibrionales; while *Bistorta* was positively correlated with Christensenellaceae, Lachnospiraceae, and unclassified_c__Clostridia, but negatively correlated with Muribaculaceae (Figure 6F).

## 4 Discussion

This study investigated the dietary diversity of zokors using the chloroplast gene *trnL*. A total of 28 families, 57 genera, and 68 plant species were identified from all stomach content samples, providing valuable insights into their feeding habits. Polygonaceae and Asteraceae were the most abundant plant families, consistent with previous reports (Zhang et al. 2021). Further analysis revealed significant differences in dietary diversity, composition, and the proportions of primary dietary components between the control and grazing groups, with only 28.99% of dietary species shared between the two groups. Animals can dynamically adjust their food intake proportions according to the availability of environmental resources (Fan et al. 2022; Guo et al. 2021). Therefore, the observed variation in zokor diets suggests that grazing may substantially influence the composition and structure of local plant communities. As dietary generalists, zokors can consume nearly all available plant species in their habitat, indicating that their dietary diversity may indirectly reflect the plant diversity in their environment (Xie et al. 2014; Zhang, Zou, et al. 2022). Thus, the higher dietary diversity observed in the grazing group implies that grazing activities could enhance vegetation diversity by suppressing the dominance of certain species, thereby potentially improving ecosystem stability (Li et al. 2021; Wang et al. 2020).

The alpha and beta diversity of microbial communities in the zokor stomach exhibited significant differences, whereas only beta diversity varied significantly in the gut. The extreme environment of the stomach, characterized by low pH and high digestive enzyme activity, restricts microbial diversity, making stomach microbial communities more sensitive to dietary fluctuations (Wu et al. 2014). Moreover, as the uppermost segment of the digestive tract, the stomach is likely more susceptible to changes in food resources. The gut (cecum), as the primary fermentation site in zokors, provides a neutral pH, stable physicochemical conditions, and complex nutrient substrates that support diverse microbial niches. Among all digestive tract segments, the cecum harbors the most diverse microbial community, potentially maintaining stable alpha diversity through functional redundancy or the stability of core microbial populations (Li et al. 2017; Mamun et al. 2020; Tinker and Ottesen 2016). Nevertheless, grazing reshaped the microbial community structure of zokors by altering the relative abundance of specific microbial taxa, similar to the findings observed in plateau pikas (Zhao et al. 2024). Notably, the relative abundance of Proteobacteria and Chloroflexi in the stomach increased significantly in the grazing group. Proliferation of Proteobacteria is often associated with host metabolic disorders (Shin et al. 2015); for instance, Proteobacteria impair antitumor immunity in mice by depleting the metabolite arginine (Meza‐Perez et al. 2024). The presence of Chloroflexi correlates with host energy metabolism, environmental adaptation, or pathological states, serving as an indicator of specific ecological adaptations or functional shifts in gut microbiota (Singh et al. 2025; Zhang, Cui, et al. 2022; Zheng et al. 2024). Probiotic families Lachnospiraceae and Christensenellaceae exhibited higher relative abundance in control groups. Lachnospiraceae degrades cellulose and hemicellulose to produce short‐chain fatty acids (Abdugheni et al. 2022; Niu et al. 2023), while Christensenellaceae is strongly linked to health and may serve as a robust health biomarker and a promising therapeutic target (Ignatyeva et al. 2023; Tavella et al. 2021). These differential microbial abundances across the zokor digestive tract likely represent adaptive responses to altered food resources. Our results suggest that livestock grazing is associated with the gastrointestinal microbiota of subterranean rodents.

The complexity of the microbial co‐occurrence network in the stomach was significantly higher in the grazing group compared to the control group. Previous studies have shown that increased network complexity enhances the resilience of microbial communities to environmental fluctuations (Roche‐Lima et al. 2018). Consistent with this, our dietary analysis revealed that zokors inhabiting grazing environments consumed more leguminous plants, which are typically rich in toxic secondary metabolites. The elevated complexity of the stomach microbial network in the grazing group may thus represent an adaptive mechanism that facilitates detoxification, as the foregut serves as a primary detoxification site in mammals (Dearing and Weinstein 2022; Zhang et al. 2018). Similarly, woodrats possess a well‐developed cecum, yet their foregut harbors a dense and metabolically active microbial community capable of degrading toxic secondary compounds ingested through the diet (Kohl and Dearing 2016; Kohl et al. 2014). In controlled dietary experiments with voles, groups fed high‐tannin diets also developed more complex microbial networks (Li, Li, Kohl, et al. 2019). Likewise, supplementing the diet of indoor‐reared plateau pikas with swainsonine significantly enhanced their gut microbial network complexity (Ren et al. 2023). As the primary fermentation site, the stomach is directly exposed to diverse plant substrates ingested during grazing, including complex carbohydrate structures and high concentrations of secondary metabolites. These heterogeneous substrates likely necessitate the development of multi‐layered cross‐regulatory metabolic pathways in microbial communities, potentially driving the formation of complex microbial co‐occurrence networks in zokor stomachs as an adaptive response to grazing‐induced pressures.

Under grazing conditions, the core functions of the microbial communities in the stomach of zokors were concentrated in the fundamental metabolic framework (Global and overview maps) and nutrient metabolism (amino acid metabolism, carbohydrate metabolism), suggesting that their microbial communities may rely more on basic metabolic pathways to maintain homeostasis. For example, they may obtain energy and growth resources through Glycolysis/Gluconeogenesis (ko00010) and lysine biosynthesis (ko00300). In contrast, although the control group also involved amino acid metabolism and carbohydrate metabolism, their enriched pathways leaned more toward specific branches (histidine metabolism ko00340, Glyoxylate and dicarboxylate metabolism ko00630), possibly reflecting microbial adaptation to the utilization of specific substrates (e.g., degrading complex carbohydrates or metabolizing particular amino acids). In the grazing group, the enrichment of the TCA cycle (ko00020), ribosome synthesis (ko03010), and lipopolysaccharide biosynthesis (ko00540) in the gut implied characteristics of efficient energy metabolism and adaptation to environmental stress. Meanwhile, the control group showed enrichment in starch and sucrose metabolism (ko00500), ABC transporters (ko02010), and the Phosphotransferase system (PTS) (ko02060), indicating microbial utilization of complex carbohydrates (such as dietary fiber or starch) in the host's diet. The differences in microbial functions between the stomach and gut of zokors may reflect responses to dietary changes. Similar functional divergences in gut microbiota driven by dietary differences have been observed in giant pandas (Wu et al. 2017), Ethiopian geladas (Baniel et al. 2021), and white‐lipped deer (Li et al. 2025).

The NCM model showed that the stochastic process of the microbiome in the stomach and gut of zokors was reduced in the grazing group, indicating diminished microbial dispersal and a greater influence of deterministic selection. These findings align with NST analyses, suggesting that strengthened deterministic processes may facilitate host adaptation to resource fluctuations by selecting for functionally specialized microbes. In contrast, gut microbial communities in both grazing and control groups remained predominantly governed by stochastic processes. The persistence of stochasticity in the gut may be attributed to its more stable physicochemical environment or a buffering effect from a larger microbial reservoir, which mitigates grazing‐induced disturbances. Meanwhile, phylogenetic null models confirmed that stochastic processes accounted for over 80% of community assembly across all groups, highlighting their foundational role in shaping the gastrointestinal microbial structure of plateau zokors. Similar patterns were observed in the gut microbiota of zebrafish (Burns et al. 2016), fish (Yan et al. 2016), and Kuruma shrimp (Zhang et al. 2024). Moreover, significant differences in five ecological processes between grazing and control groups further underscore grazing as a key driver of microbial community dynamics.

The beta diversity of both stomach and gut microbiota in zokors showed a significant positive correlation with dietary beta diversity, and similar results were observed in plateau pikas (Li et al. 2016). This indicates that variation in diet directly shapes microbial community structure, underscoring resource availability as a key driver of microbial assembly. Particularly for herbivores, plant‐derived materials serve as the primary nutritional foundation for microbiota (Gralka et al. 2020). Procrustes analysis further reinforced this association, revealing significant concordance between dietary composition and microbial community ordination. This spatial correspondence likely reflects co‐evolutionary dynamics between functional adaptations of host‐associated microbes and dietary substrates. Correlation heatmaps revealed specific plant–microbe interactions; for instance, the positive correlation between *Bistorta* and fiber‐degrading bacteria (e.g., Lachnospiraceae, Oscillospiraceae, and Christensenellaceae) suggests microbial specialization in degrading polysaccharides unique to this plant (Biddle et al. 2013; Gao et al. 2022; Xue et al. 2022). Conversely, the negative correlations between *Arctium* and several microbial taxa may result from the growth‐inhibitory effects of its secondary metabolites (Wang et al. 2019). Divergent microbial responses to the same plant across digestive compartments—such as opposing correlations between *Arctium* and Muribaculaceae in the stomach versus gut—highlight the necessity of considering regional physicochemical conditions and substrate processing stages when studying diet–microbe interactions.

## 5 Conclusion

In summary, our results highlight that grazing is associated with dietary diversity and gut microbiota of plateau zokors. Analysis revealed that zokors from grazing areas exhibited increased dietary diversity and an altered microbial community structure compared to those from non‐grazing areas, characterized by lower abundance of beneficial taxa (e.g., Lachnospiraceae, Christensenellaceae) and elevated levels of potential pathogens (Proteobacteria). Increased complexity of stomach microbial co‐occurrence networks and functional differentiation of the microbiota were also detected. Although stochastic processes primarily governed microbial community assembly, their influence diminished under grazing, indicating a shift toward deterministic processes. Furthermore, significant associations were identified between specific dietary plants and microbial taxa, indicating a strong diet–microbiota relationship. These findings indicate substantial variation in the dietary niches and gut microbial ecology of subterranean rodents across grazing regimes, providing new insights for ecosystem management and conservation.

## Author Contributions

**Shien Ren:** data curation (equal), investigation (equal), methodology (equal), visualization (equal), writing – original draft (equal). **Jing Yang:** investigation (equal), methodology (equal), visualization (equal). **Xiaoluo Aba:** data curation (equal), investigation (equal). **Yang Hu:** data curation (equal), investigation (equal). **Yifan Zhao:** data curation (equal), investigation (equal). **Shoushu Pang:** investigation (equal). **Chongxuan Han:** investigation (equal). **Liangzhi Zhang:** conceptualization (equal), funding acquisition (equal), writing – review and editing (equal). **Xiaoning Nan:** conceptualization (equal), funding acquisition (equal), methodology (equal), project administration (equal), supervision (equal), writing – review and editing (equal).

## Ethics Statement

All experimental procedures were approved by the Ethics Committee of Northwest A&F University and adhered to institutional animal welfare regulations.

## Conflicts of Interest

The authors declare no conflicts of interest.

## Supporting information


**Appendix S1:** ece372377‐sup‐0001‐AppendixS1.pdf.

**Appendix S2:** ece372377‐sup‐0002‐AppendixS2.zip.

## Data Availability

All the required data are uploaded as Supporting Information.
