# Supplementary material for: Grazing Is Associated With Dietary Diversity and Gastrointestinal Microbiota in Subterranean Rodents
Source: Ecol Evol. 2025 Nov 2;15(11):e72377. doi: 10.1002/ece3.72377 (PMC12579973; doi:10.1002/ece3.72377)
Supplement: Supplementary file 1 — Appendix S1: ece372377‐sup‐0001‐AppendixS1.pdf. [file ECE3-15-e72377-s002.pdf]

Grazing is associated with dietary diversity and gastrointestinal microbiota in subterranean rodents

Shien Ren [1], Jing Yang [2], Xiaoluo Aba [1], Yang Hu [1], Yifan Zhao [1], Shoushu Pang [2], Chongxuan Han [1], Liangzhi Zhang [3, *], Xiaoning Nan [1, *]

1 Key Laboratory of National Forestry and Grassland Administration on Management of Western Forest Bio-Disaster, College of Forestry, Northwest A&F University, Yangling, Shaanxi, 712100, China

2 Institute of Bailongjiang Forestry Science of Gansu Province, Lanzhou, 730070, China

3 Key Laboratory of Adaptation and Evolution of Plateau Biota, Northwest Institute of Plateau Biology, Chinese Academy of Sciences, Xining, 810008, China

Shien Ren and Jing Yang contributed equally to this work.

*Correspondence: Liangzhi Zhang (lzzhang@nwipb.cas.cn); Xiaoning Nan (nxn@nwsuaf.edu.cn)

Supporting files,

Table S1-S7

Figure S1-S5

Table S1. The detailed information of sample collection.

Sample ID	Elevation (m)	Geographic coordinates
Control_1	3466	103.41°E, 34.94°N
Control_2	3466	103.41°E, 34.94°N
Control_3	3466	103.41°E, 34.94°N
Control_4	3400	103.38°E, 34.94°N
Control_5	3400	103.38°E, 34.94°N
Control_6	3400	103.38°E, 34.94°N
Control_7	3303	103.34°E, 34.93°N
Control_8	3303	103.34°E, 34.93°N
Control_9	3303	103.34°E, 34.93°N
Grazing_1	3411	103.40°E, 35.02°N
Grazing_2	3411	103.40°E, 35.02°N
Grazing_3	3411	103.40°E, 35.02°N
Grazing_4	3468	103.43°E, 35.01°N
Grazing_5	3468	103.43°E, 35.01°N
Grazing_6	3468	103.43°E, 35.01°N
Grazing_7	3437	103.47°E, 35.01°N
Grazing_8	3437	103.47°E, 35.01°N
Grazing_9	3437	103.47°E, 35.01°N

Table S2. Differences in the relative abundance of dominant families, genera and species in the diet of plateau zokors between control and grazing groups.

Taxa	Control-Mean	Grazing-Mean	p-adjust
**Families**			
Polygonaceae	0.808819	0.219035	0.010
Asteraceae	0.002736	0.265030	0.011
Apiaceae	0.000006	0.161784	0.002
Fabaceae	0.001509	0.111235	0.105
Asparagaceae	0.108263	0.001322	0.035
Ranunculaceae	0.010507	0.088769	0.074
Rosaceae	0.001849	0.083802	0.011
Cyperaceae	0.044885	0.012331	0.930
Boraginaceae	0.000000	0.036179	0.011
Brassicaceae	0.003804	0.004647	0.440
**Genera**			
Bistorta	0.775257	0.117643	0.005
Arctium	0.002736	0.264900	0.013
Carum	0.000000	0.151855	0.002
Cyamopsis	0.000000	0.111031	0.495
Polygonatum	0.108263	0.001322	0.035
Ranunculus	0.001771	0.084745	0.013
Potentilla	0.001479	0.083802	0.009
Rumex	0.026598	0.047130	0.495
Carex	0.044221	0.012331	0.879
Koenigia	0.001129	0.046014	1.000
**Species**			
Bistorta vivipara	0.821545	0.146374	0.007
Arctium lappa	0.002839	0.238601	0.013
Carum carvi	0.000000	0.136670	0.003
Cyamopsis tetragonoloba	0.000000	0.099928	0.495
Polygonatum stenophyllum	0.121795	0.001190	0.046
Ranunculus cassubicus	0.000074	0.077606	0.010
Potentilla micropetala	0.000003	0.076144	0.007
Rumex alpinus	0.000226	0.066174	0.495
Carex maritima	0.016661	0.037525	0.931

Koenigia nepalensis	0.000000	0.041048	0.129
Asperugo procumbens	0.000000	0.035342	0.068
Rheum pumilum	0.005766	0.005066	0.102
Aegopodium podagraria	5.51E-06	0.009893	0.010
Viola biflora	0.005821	0.000000	0.007
Anemone trullifolia	0.005171	0.000000	0.006
Ajuga lupulina	0.000000	0.004758	0.495
Descurainia sophia	2.75E-06	0.004645	1.000
Deschampsia cespitosa	0.001129	0.002678	1.000
Cardamine macrophylla	0.003802	2.75E-06	0.347
Platanthera hyperborea	0.001253	0.002055	1.000

Table S3. Differences in the relative abundance of dominant phyla and families in the stomach microbiota of plateau zokors between the SC and SG groups.

**Taxa**	**SC-Mean**	**SG-Mean**	**p-adjust**
**Phyla**			
Firmicutes	0.610227	0.706599	0.419
Bacteroidota	0.293372	0.148141	0.104
Proteobacteria	0.016860	0.080401	0.043
Desulfobacterota	0.052013	0.026329	0.128
Actinobacteriota	0.019596	0.021767	0.536
Fusobacteriota	0.000048	0.005931	0.040
Acidobacteriota	0.000385	0.003259	0.045
Chloroflexi	0.000670	0.002496	0.040
Spirochaetota	0.002540	0.000035	0.154
Cyanobacteria	0.000914	0.000828	0.250
**Families**			
Lactobacillaceae	0.096808	0.589666	0.010
Muribaculaceae	0.292153	0.139599	0.052
Lachnospiraceae	0.261173	0.015974	< 0.001
Oscillospiraceae	0.095910	0.014014	0.009
Ruminococcaceae	0.069684	0.019166	0.013
Pasteurellaceae	0.011176	0.065485	0.023
Desulfovibrionaceae	0.048189	0.023759	0.074
Streptococcaceae	0.009004	0.030428	0.158
Christensenellaceae	0.034428	0.004555	0.005
Eggerthellaceae	0.012321	0.006579	0.019
norank_o__Clostridia_UCG-014	0.010053	0.003625	0.013
Peptostreptococcaceae	0.000834	0.011269	0.009
Monoglobaceae	0.008840	0.002085	0.013
Anaerovoracaceae	0.008908	0.001091	0.005
unclassified_o__Desulfovibrionales	0.003692	0.002277	0.120

Table S4. Differences in the relative abundance of dominant phyla and families in the gut microbiota of plateau zokors between the GC and GG groups.

Taxa	GC-Mean	GG-Mean	p-adjust
**Phyla**			
Firmicutes	0.794737	0.529913	0.015
Bacteroidota	0.164856	0.406706	0.010
Desulfobacterota	0.027827	0.055272	0.128
Actinobacteriota	0.004975	0.004061	1.000
Spirochaetota	0.004776	0.000051	0.314
Proteobacteria	0.000382	0.000664	0.085
Patescibacteria	0.000308	0.000574	0.662
Cyanobacteria	0.000225	0.000311	0.662
Elusimicrobiota	0.000016	0.000103	0.197
Chloroflexi	0.000000	0.000080	0.085
**Families**			
Lachnospiraceae	0.471934	0.209343	0.025
Muribaculaceae	0.162659	0.404429	0.015
Oscillospiraceae	0.182049	0.146781	0.263
Ruminococcaceae	0.072298	0.099894	1.000
Desulfovibrionaceae	0.026082	0.050377	0.165
Christensenellaceae	0.017187	0.005087	0.056
Eubacterium_coprostanoligenes_group	0.004847	0.013184	0.165
UCG-010	0.006188	0.010816	0.210
norank_o__Clostridia_UCG-014	0.007747	0.008885	0.905
Monoglobaceae	0.007795	0.007358	0.921
unclassified_c__Clostridia	0.007439	0.004456	0.025
norank_o__Clostridia_vadinBB60_group	0.004568	0.005703	0.921
Anaerovoracaceae	0.003487	0.004821	0.813
Eggerthellaceae	0.004423	0.002903	0.565
unclassified_o__Desulfovibrionales	0.001739	0.004882	0.102

Table S5. Network indices of the stomach and gut microbiota of plateau zokors.

**Network indices**	**SC**	**SG**	**GC**	**GG**
Total nodes	45	48	47	48
Total links	96	342	157	167
Positive links	58	215	89	97
Negative links	38	127	68	70
Average degree	4.267	14.250	6.681	6.958
Density	0.097	0.303	0.145	0.148
Average clustering coefficient	0.475	0.728	0.505	0.566
Average path length	2.987	1.980	2.972	2.755

Table S6. LEfSe analysis results of KEGG level 3 functions of the stomach microbiota in plateau zokors.

Pathway level 2	Pathway ID	Level 3 description	LDA score (log10)	p-adjust
Cell motility	ko02040	Flagellar assembly	3.33	< 0.001
Metabolism of cofactors and vitamins	ko00860	Porphyrin and chlorophyll metabolism	3.28	< 0.001
Cell motility	ko02030	Bacterial chemotaxis	3.27	< 0.001
Signal transduction	ko02020	Two-component system	3.18	0.006
Global and overview maps	ko01230	Biosynthesis of amino acids	3.11	0.011
Cellular community - prokaryotes	ko02024	Quorum sensing	3.07	< 0.001
Global and overview maps	ko01210	2-Oxocarboxylic acid metabolism	2.85	0.004
Amino acid metabolism	ko00400	Phenylalanine, tyrosine and tryptophan biosynthesis	2.85	0.004
Energy metabolism	ko00910	Nitrogen metabolism	2.83	0.004
Carbohydrate metabolism	ko00040	Pentose and glucuronate interconversions	2.76	0.004
Cellular community - prokaryotes	ko02026	Biofilm formation - Escherichia coli	2.73	0.004
Carbohydrate metabolism	ko00630	Glyoxylate and dicarboxylate metabolism	2.71	0.038
Amino acid metabolism	ko00290	Valine, leucine and isoleucine biosynthesis	2.70	0.004
Cell growth and death	ko04217	Necroptosis	2.62	0.004
Amino acid metabolism	ko00340	Histidine metabolism	2.62	0.005
Biosynthesis of other secondary metabolites	ko00940	Phenylpropanoid biosynthesis	2.58	0.006
Metabolism of terpenoids and polyketides	ko00523	Polyketide sugar unit biosynthesis	2.53	0.006
Immune system	ko04621	NOD-like receptor signaling pathway	2.51	0.004
Glycan biosynthesis and metabolism	ko00550	Peptidoglycan biosynthesis	2.51	0.032
Amino acid metabolism	ko00350	Tyrosine metabolism	2.53	0.004
Replication and repair	ko03410	Base excision repair	2.54	0.005
Metabolism of cofactors and vitamins	ko00790	Folate biosynthesis	2.54	0.009

Energy metabolism	ko00680	Methane metabolism	2.54	0.006
Drug resistance: antimicrobial	ko01503	Cationic antimicrobial peptide (CAMP) resistance	2.57	< 0.001
Translation	ko00970	Aminoacyl-tRNA biosynthesis	2.57	0.014
Signal transduction	ko04066	HIF-1 signaling pathway	2.60	0.021
Nucleotide metabolism	ko00240	Pyrimidine metabolism	2.62	0.004
Endocrine system	ko04922	Glucagon signaling pathway	2.64	0.025
Replication and repair	ko03030	DNA replication	2.65	0.004
Amino acid metabolism	ko00260	Glycine, serine and threonine metabolism	2.67	0.006
Infectious disease: bacterial	ko05150	Staphylococcus aureus infection	2.75	0.004
Global and overview maps	ko01220	Degradation of aromatic compounds	2.75	0.004
Amino acid metabolism	ko00300	Lysine biosynthesis	2.80	0.009
Cancer: overview	ko05230	Central carbon metabolism in cancer	2.87	0.006
Metabolism of other amino acids	ko00480	Glutathione metabolism	2.88	0.004
Global and overview maps	ko01200	Carbon metabolism	2.90	0.006
Carbohydrate metabolism	ko00620	Pyruvate metabolism	2.93	0.004
Metabolism of cofactors and vitamins	ko00130	Ubiquinone and other terpenoid-quinone biosynthesis	2.97	0.004
Global and overview maps	ko01110	Biosynthesis of secondary metabolites	2.97	0.021
Nucleotide metabolism	ko00230	Purine metabolism	3.02	0.004
Membrane transport	ko02060	Phosphotransferase system (PTS)	3.08	0.025
Carbohydrate metabolism	ko00010	Glycolysis / Gluconeogenesis	3.09	0.004
Translation	ko03010	Ribosome	3.11	0.005
Global and overview maps	ko01120	Microbial metabolism in diverse environments	3.14	0.004

Table S7. LEfSe analysis results of KEGG level 3 functions of the gut microbiota in plateau zokors.

**Pathway level 2**	**Pathway ID**	**Level 3 description**	**LDA score (log10)**	**p-adjust**
Membrane transport	ko02010	ABC transporters	3.36	0.005
Carbohydrate metabolism	ko00500	Starch and sucrose metabolism	2.91	0.026
Cellular community - prokaryotes	ko02024	Quorum sensing	2.82	0.005
Metabolism of cofactors and vitamins	ko00860	Porphyrin and chlorophyll metabolism	2.82	< 0.001
Carbohydrate metabolism	ko00030	Pentose phosphate pathway	2.65	0.010
Membrane transport	ko02060	Phosphotransferase system (PTS)	2.63	0.015
Translation	ko03010	Ribosome	2.54	0.031
Glycan biosynthesis and metabolism	ko00540	Lipopolysaccharide biosynthesis	2.61	0.005
Carbohydrate metabolism	ko00020	Citrate cycle (TCA cycle)	2.64	0.015
Energy metabolism	ko00720	Carbon fixation pathways in prokaryotes	2.72	0.015

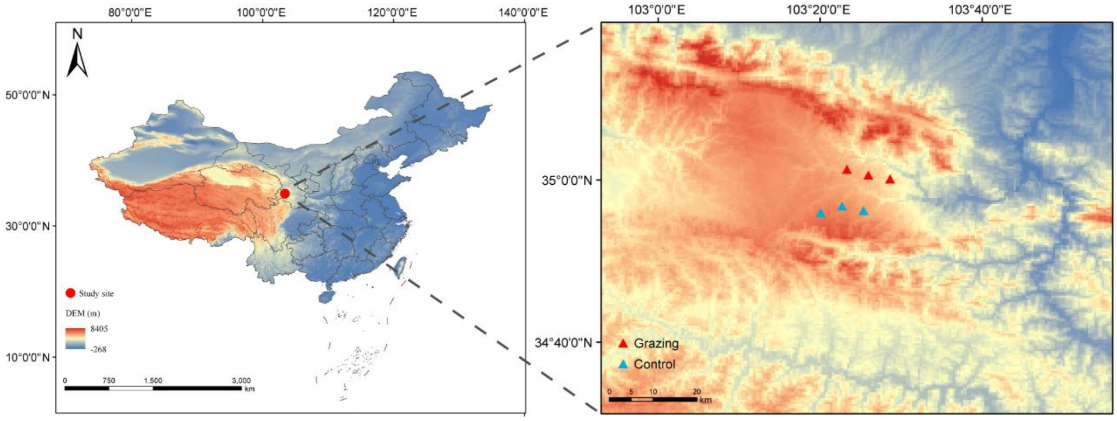

Figure S1 Locations of the study site. Based on the standard map supervised by the Ministry of Natural Resources of the People's Republic of China [No. GS (2023) 2763].

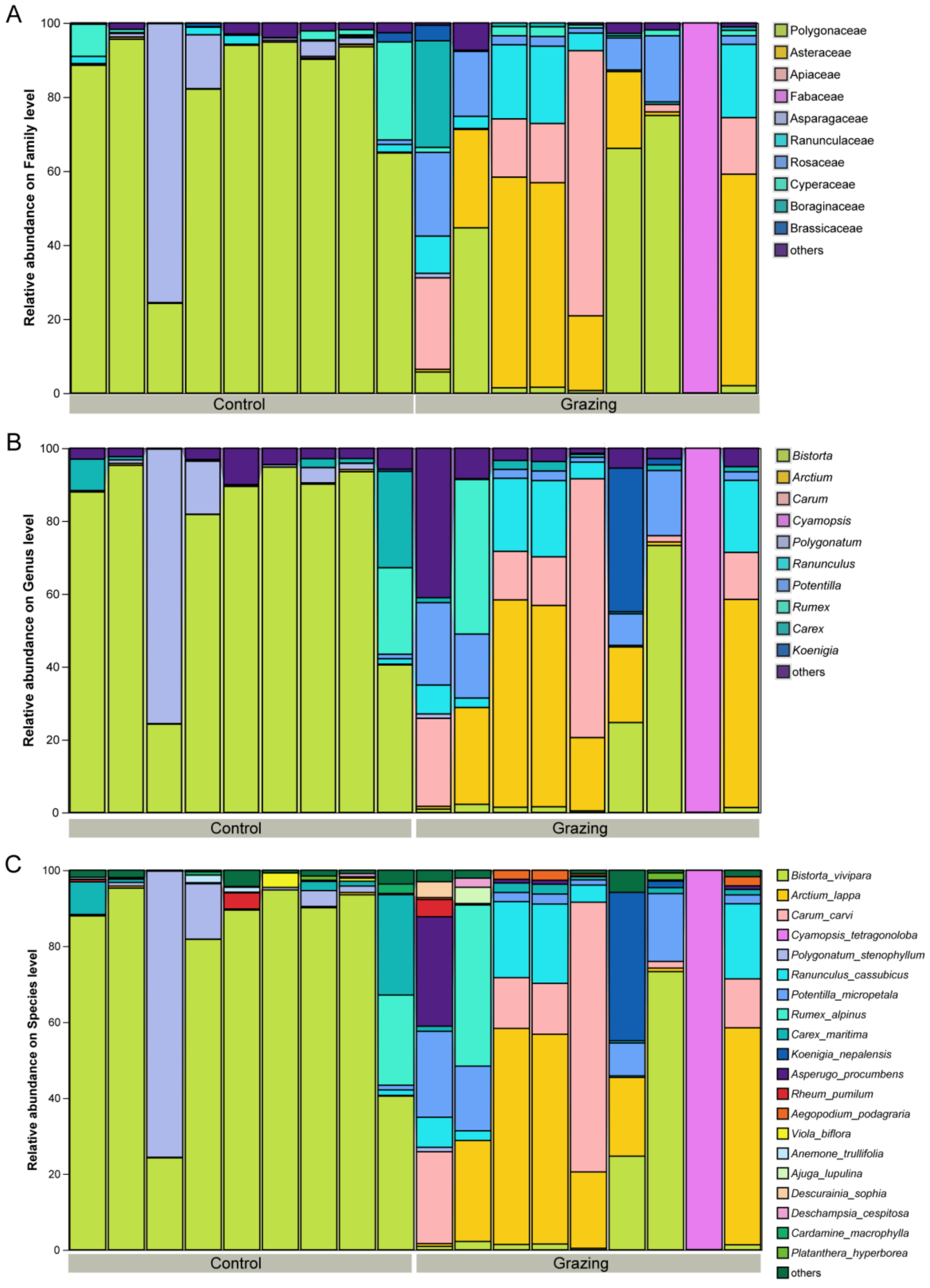

Figure S2 Diet composition of plateau zokors. (A) Family-level, (B) genus-level, and (C) species-level diet composition.

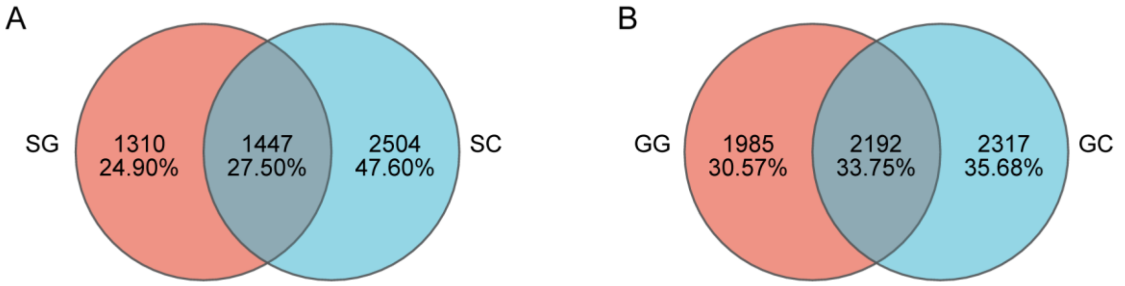

Figure S3 Venn diagrams depicting the composition of the gastrointestinal microbiota in plateau zokors. Venn diagrams of the (A) stomach and (B) gut microbiota composition.

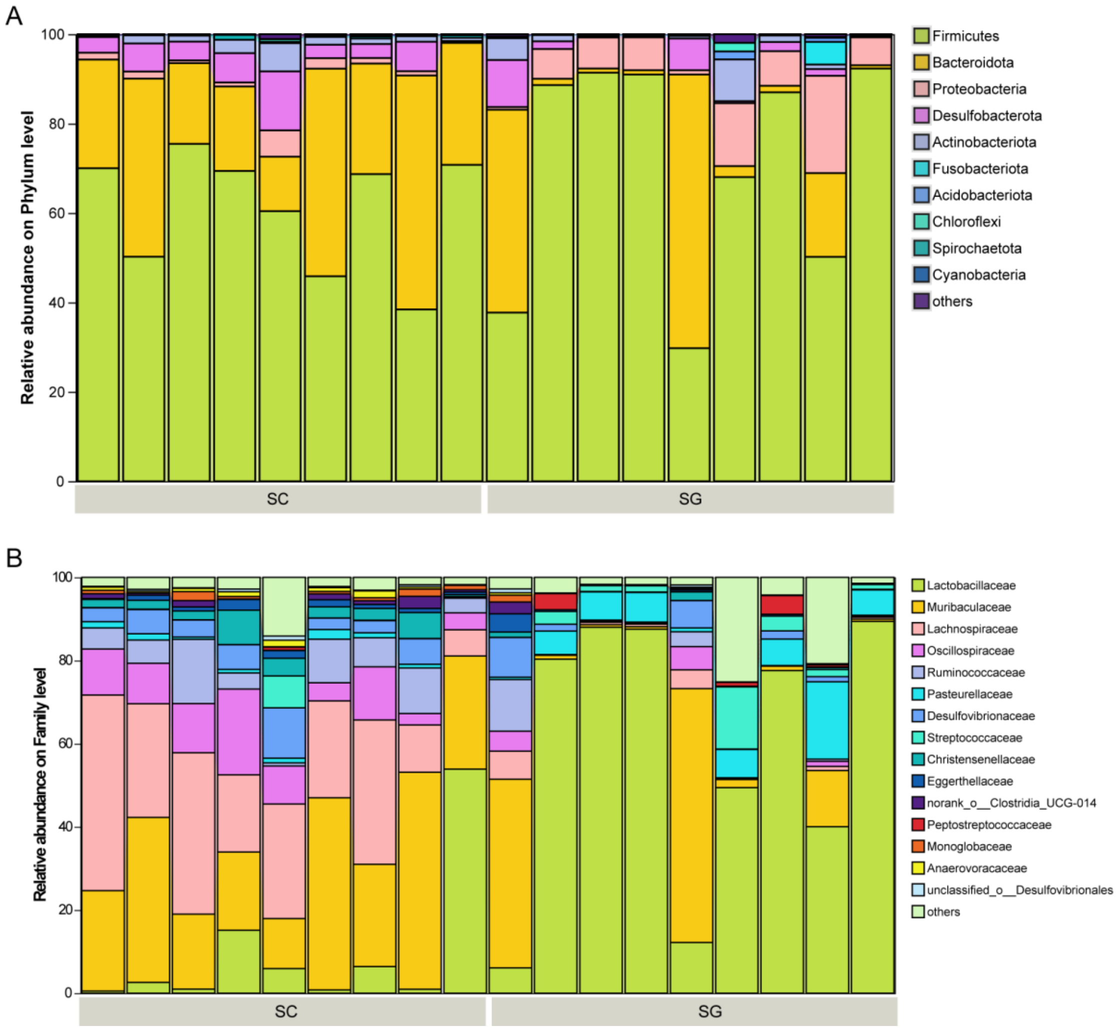

Figure S4 Composition of stomach microbiota in plateau zokors. (A) At the phylum level. (B) At the family level.

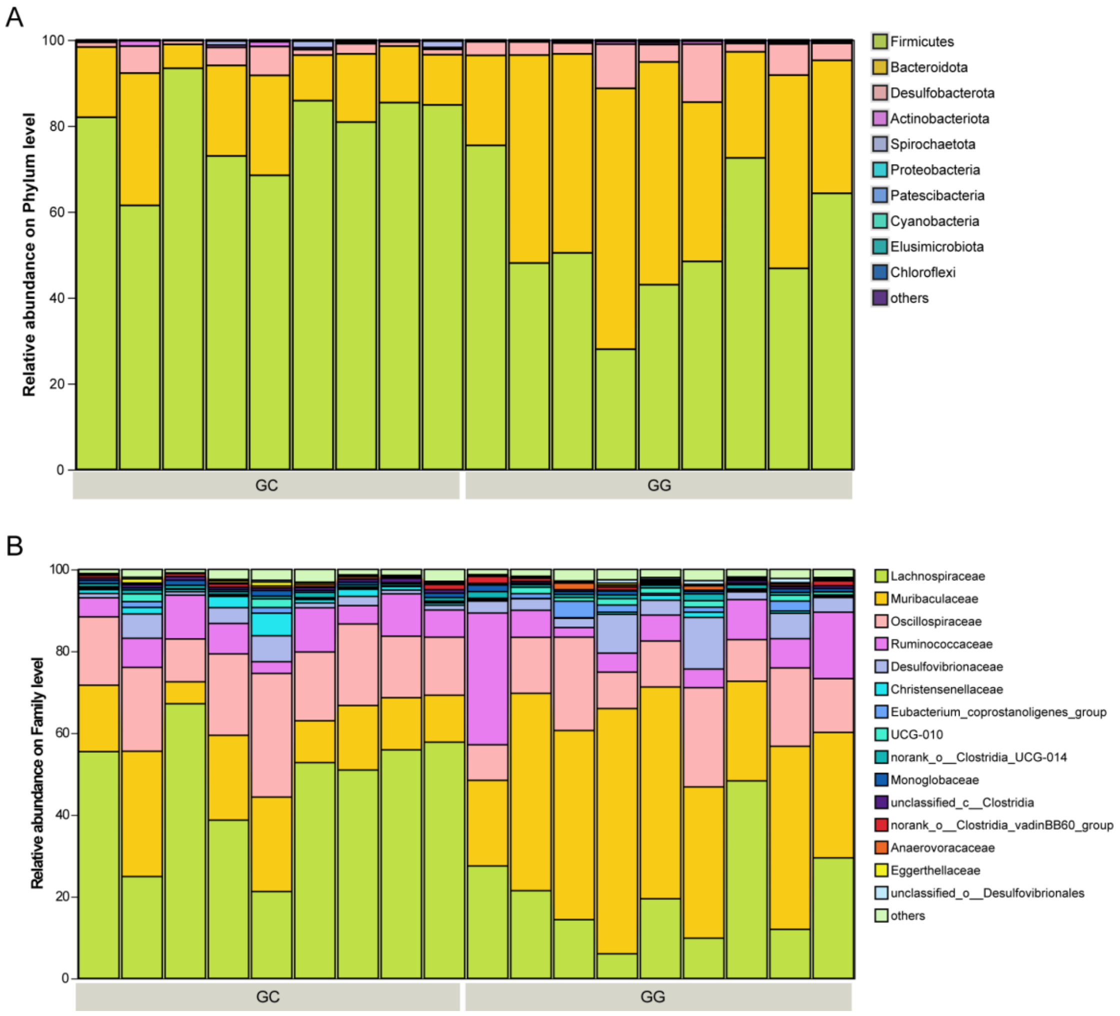

Figure S5 Composition of gut microbiota in plateau zokors. (A) At the phylum level. (B) At the family level.
