# Supplementary material for: Grazing Is Associated With Dietary Diversity and Gastrointestinal Microbiota in Subterranean Rodents
Source: Ecol Evol. 2025 Nov 2;15(11):e72377. doi: 10.1002/ece3.72377 (PMC12579973; doi:10.1002/ece3.72377)
Supplement: Supplementary file 2 — Appendix S2: ece372377‐sup‐0002‐AppendixS2.zip. [file ECE3-15-e72377-s001.zip › Raw data.docx]

The sequencing raw data can be accessed through the link below.

<https://pan.baidu.com/s/1I9EaD7uq10PGrwKlPY5Fwg?pwd=vo0h>
